# Breaking the Tradeoff between Oil Film Thickness and Viscous Friction: *n*-Alcohol-Containing Lubricants in High-Pressure Contacts

**DOI:** 10.1021/acsami.4c22374

**Published:** 2025-03-17

**Authors:** Tom Reddyhoff, Wren Montgomery, Muhammad Aqif Suhaimee, Pushkar Deshpande, Yunhao Xia, Peng Wang, James Ewen

**Affiliations:** † Department of Mechanical Engineering, Imperial College London, Exhibition Road, South Kensington, London SW7 2AZ, U.K.; ‡ Science Innovation Platform, The Natural History Museum, Cromwell Road, South Kensington, London SW7 5BD, U.K.

**Keywords:** elastohydrodynamic lubrication, EHL, friction, viscosity, film thickness, superlubricity, alcohols, polymorphism

## Abstract

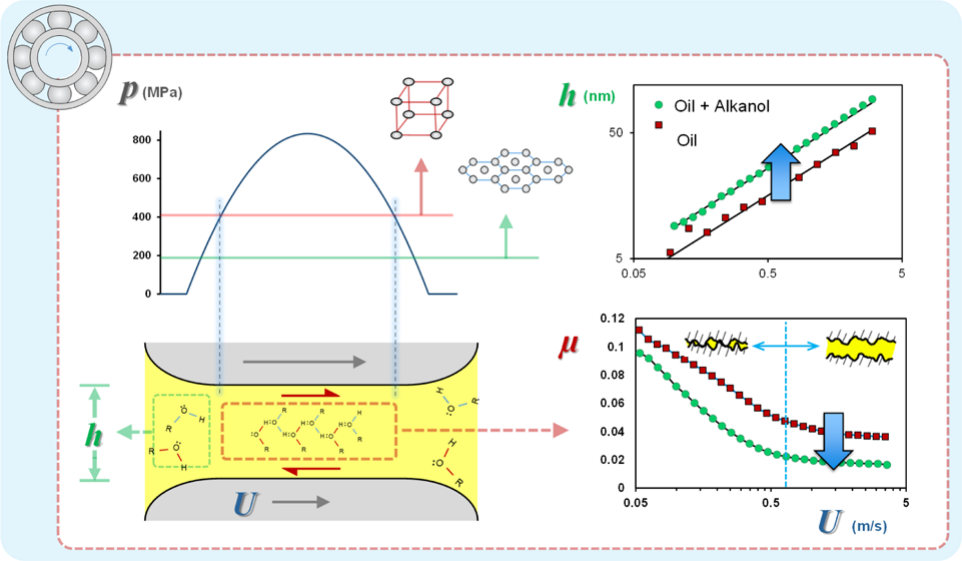

Low-friction lubricant formulations are urgently needed to improve the energy efficiency of machines. Here, we show that blending 1-dodecanol with a hydrocarbon oil improves lubrication in nonconformal sliding/rolling contacts by simultaneously increasing hydrodynamic film thickness and reducing viscous friction. This is due to pressure-induced polymorphic phase transformations in the 1-dodecanol molecules after they flow through the film-thickness-determining inlet and reach the load-supporting zone. At relatively low pressures, 1-dodecanol forms a lamellar hexagonal solid polymorph that gives durable superlubricity and then, at higher pressures, it forms an orthorhombic polymorph. Both polymorphs cause anomalously low friction when blended into various hydrocarbon base oils over a wide range of speed, pressure, and shear rate conditions representative of rolling bearing and gear contacts. By breaking the ubiquitous tradeoff between friction and film thickness and enabling superlubricity, these blends pave the way for considerable energy efficiency improvements in widespread lubricated contacts.

## Introduction

Approximately 23% of global energy consumption originates from friction and wear in tribological contacts.^1^ Reducing friction and wear through improved lubricant chemistry is, therefore, one of the most generic and productive ways to reduce fuel consumption and greenhouse gas emissions in the pursuit of net zero.

Typical lubricants consist of a hydrocarbon base oil, into which a package of additives is dissolved, usually at a ratio of ∼10:1.^2^ The key property of a lubricant base oil is its viscosity, which enables it to generate hydrodynamic lift to separate the sliding surfaces, thus minimizing solid–solid contact. This fluid film also dissipates energy through viscous drag, particularly at high speeds. Constrained by this tradeoff,^3^ reduced friction in liquid-lubricated contacts is typically achieved through three approaches. First, the viscosity of the base oil can be reduced to lower viscous friction.^4,5^ However, this method is limited since excessively reduced viscosity leads to liquid lubricant films that are too thin to separate the sliding surfaces. This results in increased solid–solid contact, higher friction, more wear and surface damage, and shorter component lives.^6^ To partially mitigate this, surface-active friction modifier additives can be blended with liquid lubricants to form low-shear strength interfacial layers on components that reduce friction when there is insufficient hydrodynamic entrainment to produce a separating film.^7^ These friction modifier additives are included in most lubricating oils but are only effective under nonoptimal conditions—specifically when opposing surfaces come into contact. Furthermore, these additives must function alongside many other surface-active additives and be effective even on rough components, both of which can hinder their performance. A third approach is to blend high-molecular-weight viscosity modifier additives into the base oil.^8^ These polymers increase viscosity under low shear conditions to give a thicker entrained lubricant film to separate surfaces but undergo local ordering of molecules that lowers the viscosity, i.e., shear thinning, when subject to high shear rates in the contact, where the gap between surfaces is thinner. However, this mechanism is limited because the viscosity can never be reduced below that of the base oil. Moreover, viscosity modifiers are susceptible to damage, resulting in permanent viscosity loss.^9^

Here, we report a new method for modifying a lubricant to simultaneously increase its film-thickness-forming ability while reducing its viscous drag, thus breaking the tradeoff between these two parameters. The method uses 1-dodecanol as an additive, whose inclusion in the lubricant results in an apparent viscosity that is anomalously sensitive to applied pressure. This applies to high-pressure elastohydrodynamic (EHL) bearing contacts, where the conditions that determine wear-reducing film thickness differ from those that determine viscous dissipation. Such contact conditions are present in rolling/sliding components such as bearings and gears, with an estimated 50 billion of these components currently in operation throughout the world.^10^ In a typical internal combustion engine passenger car, the energy loss due to friction in high-pressure contacts is estimated to consume around 5.9% of the vehicle’s total fuel energy,^11^ which is a significant quantity, considering that these vehicles are typically only ∼20% efficient. Furthermore, the relative proportion of EHL losses increases with the transition to hybrid and electric vehicles.^12^

These blends of base oil and dodecano are also shown herein, under certain conditions, to exhibit structural, macroscale liquid superlubricity (i.e., friction that effectively vanishes, accompanied by essentially zero wear). This is important, as superlubricity may be instrumental in enabling carbon neutrality^13^ and is hence the subject of intense research efforts aimed at uncovering its fundamental mechanisms^14^ and achieving macroscale applicability.^15^ An underlying mechanism for such low friction, based on a structural mismatch between sliding atomic layers, was predicted by Hirano^16^ and Sokoloff^17^ in the 1990s. Initially, superlubricity was demonstrated in nanoscale contacts lubricated by two-dimensional materials such as mica,^18^ graphite,^19^ molybdenum disulfide,^20^ graphene,^21^ and graphene/boron nitride.^22^ In these examples, structural superlubricity^23^ results from an interlayer sliding of lamellar surfaces that are incommensurate due to either a misalignment angle^18^ or an intrinsic lattice mismatch between adjacent layers.^24^ Recently, superlubricity has been achieved in macroscale contacts, mostly under boundary lubrication conditions, thanks to the mechanochemical decomposition of molecules to passivate sliding surfaces and promote the formation of easily sheared structures. As noted by Du et al.,^13^ macroscale liquid superlubricity typically requires either nonatmospheric environments^25,26^ or submersion in liquids such as water,^27^ acid-based solutions,^28−30^ polyol aqueous solutions,^31−33^ and ionic liquids.^34,35^ These approaches come with general disadvantages, such as the requirement of prolonged running-in periods^13^ or extensive preparation, as with grafted polymer brushes on textured surfaces.^36^ More problematically, the specific environments required are not directly applicable to existing machine components, where energy is being lost. To our knowledge, it is only through the application of long-chain alcohols (alkanols), such as n-dodecanol, that self-replenishing structural superlubricity can be achieved within the bulk of an oil film, as found within common bearing contacts, without surface modification. This was shown previously for pure 1-dodecanol^37^ and is now demonstrated when it is blended with a hydrocarbon base oil.

## Background

In rolling/sliding components such as rolling bearings, gears, and cams, contact areas between surfaces are typically hundreds of micrometers in diameter and result in transient pressures on the order of gigapascals. When lubricated by piezoviscous fluids, such as hydrocarbon-based oils, these conditions give rise to classical elastohydrodynamic lubrication, EHL,^38^ whereby elastic deformation of the component surfaces and a large increase in oil viscosity lead to a significantly thicker lubricant film than in (rigid isoviscous) hydrodynamic lubrication.

When an oil enters an EHL contact, its viscosity increases by orders of magnitude due to the high contact pressures. At such high viscosities, only a very shallow film thickness gradient is needed to generate sufficient hydrodynamic pressure to balance the applied load; therefore, the gap between surfaces in the central region is approximately constant. Also, due to negligible side leakage, the film thickness in this central zone depends only upon the lubricant viscosity in the inlet region, where pressures are relatively low at around 70 MPa.^39^ According to the widely used Dowson–Hamrock equation (obtained by numerically solving the Reynolds equation while accounting for piezo viscosity and elastic surface deformation^40^), the central film thickness, *h*_c_, in the contact is given by
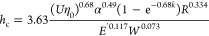
1where *U* is the average speed of the surfaces, η_0_ is the viscosity at atmospheric pressure, α is the pressure viscosity coefficient, *E*′ is the effective elastic modulus, *W* is the applied load, and *k* (≈1) is the ellipticity parameter.

In contrast to the film thickness, EHL friction (or traction) depends on lubricant properties within the contact region, where average pressures are typically in the gigapascal range. This friction is given by the integral of the shear stress, τ, over the contact area, which can be estimated using the well-known Johnson and Tevaarwerk equation (obtained by considering the energy barrier overcome as one molecular unit passes another) and neglecting the low-speed elastic contribution^41^
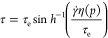
2where γ is the strain rate, τ_e_ is the “Eyring stress”^42^ at which shear thinning becomes significant, and *η*(*p*) is the effective viscosity of the oil at the prevailing contact pressure, *p* (which can be approximated by the applied normal force divided by the Hertz contact area). If it is assumed (as is approximately the case for conventional lubricants^43^) that the viscosity in the contact increases exponentially with pressure, eq 2 becomes
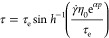
3where *η*_0_ is the viscosity at atmospheric pressure and α is the pressure viscosity coefficient. Note that this is a cursory summary of EHL friction for assessing anomalous alkanol behavior; for a more rigorous review of this area, see ref (39).

So, for a conventional lubricant, reducing oil viscosity, η_0_, reduces friction according to eq 3 (beneficial) but also gives thinner lubricant films according to eq 1 (problematic). This tradeoff may be sidestepped by using a lubricant that undergoes a pressure-induced phase change that reduces its effective fluid film viscosity (η_0_ in eq 3) when subjected to high-pressure conditions within an EHL contact. This viscosity reduction must occur after the lubricant has entered the contact so that film thickness, which depends on low-pressure viscosity η_0_ in eq 1 and thus durability, is not adversely affected.

**Figure 1:**
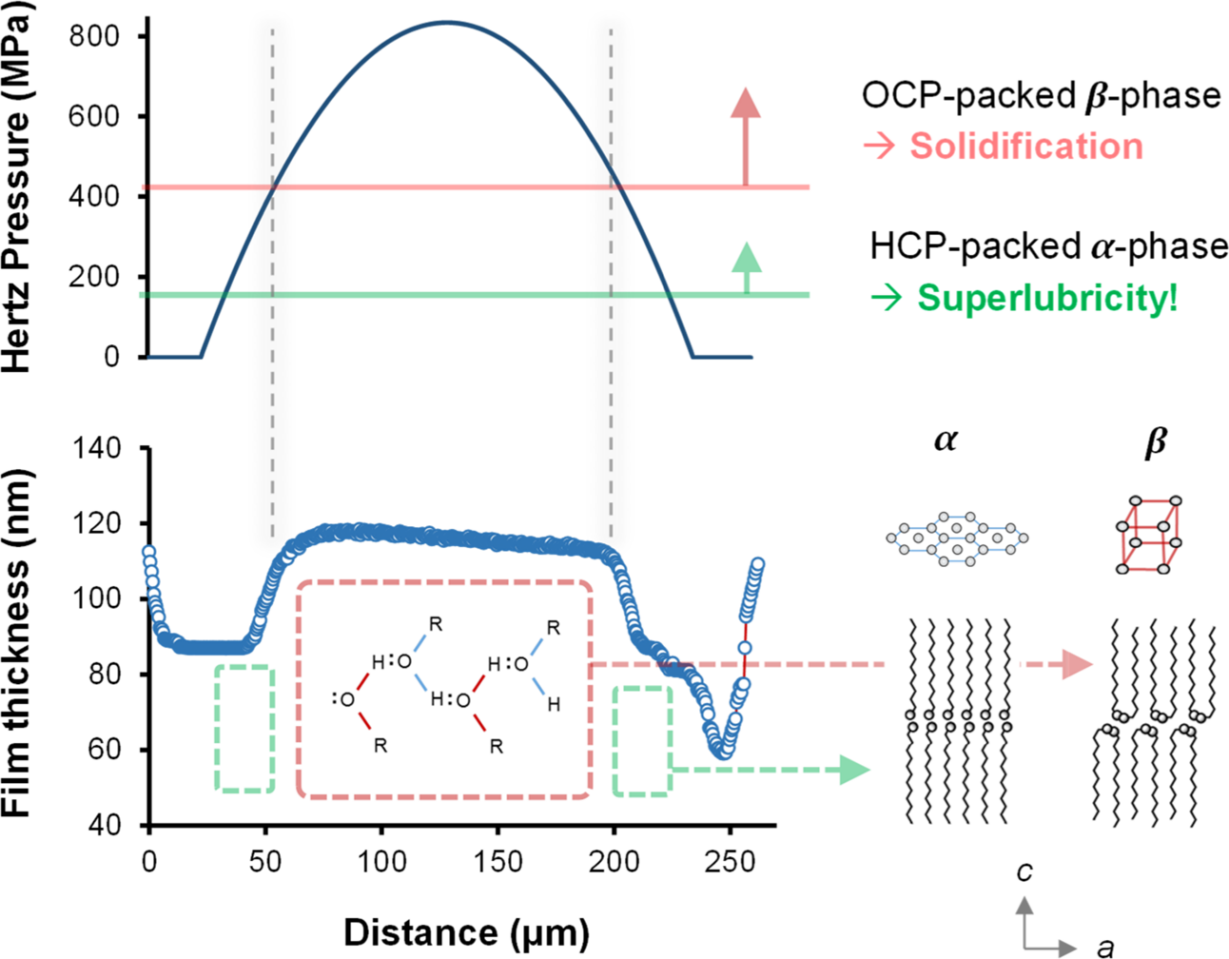
Schematic diagram showing pressure-induced polymorph formation in alkanol-lubricated contact (adapted from refs (37,54)(54)).

Dodecanol is known to behave abnormally in EHL contacts.^44−49^ This has recently been attributed to its ability to freeze into two different molecular arrangements, or polymorphs, when subject to increased pressure or reduced temperature; i.e., when liquid dodecanol is cooled or subject to increasing pressure, it first forms a very low-friction hexagonally packed α-phase (sometimes called the rotator phase^50^); then, on further cooling or increasing pressure, it forms an orthorhombic solid β-phase with high friction.^37,51,52^ Fourier transform infrared (FTIR) spectroscopy and dynamic scanning calorimetry (DSC) measurements suggest that these polymorphs form due to pressure-induced hydrogen bonding.^37^ When entrained into an elastohydrodynamic bearing contact, where the pressure varies from atmospheric to gigapascal levels, this behavior results in a bifurcation of both friction and film thickness, depending on whether the local pressure is above or below the phase transformation threshold (Figure 1). A dimple of increased film thickness occurs,^44−48^ where the solid orthorhombic phase reduces fluid flow velocity, requiring the surfaces to deform to conserve the flow of lubricant through the contact.^53^ This is an anomalous case in which the film thickness is significantly affected by conditions within the contact rather than being predetermined at the inlet. The overall friction from the two regions can be predicted using Hertz analysis (see ref (37) for derivation). This assumes that the alkanol has a constant friction value, μ_L_, at pressures below the critical pressure, *p*_H_, and another value, μ_H_, above *p*_H_ (Figure 1). These friction coefficients are weighted according to the fraction of the total load, *W*, supported by each region, which is found by integrating the corresponding regions of the Hertz pressure distribution. The overall friction coefficient is then
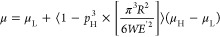
4where *E*′ and *R* are the elastic modulus and reduced radius of the surfaces, respectively.^37^

In summary, the contrasting EHL mechanisms of conventional oils and long-chain alcohols are well established. However, mixtures of these fluids have not been tested previously in EHL contacts. Of most interest is the effect of mixing on viscosity since this property affects film thickness and friction. A starting point from the extensive literature on mixtures may be the simple formulation by Grunberg and Nissan^55^ to assess how molecular interactions affect viscosity
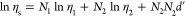
5where η_s_, η_1_, and η_2_ are the viscosities of the blend and components 1 and 2, respectively, mixed according to mole fractions, *N*_1_ and *N*_2_, while *d*′ is an interaction parameter, which is positive if interactions between unlike molecules are strong and conversely negative if these interactions are weak.^56^

## Results

### Anomalous Friction Behavior

**Figure 2:**
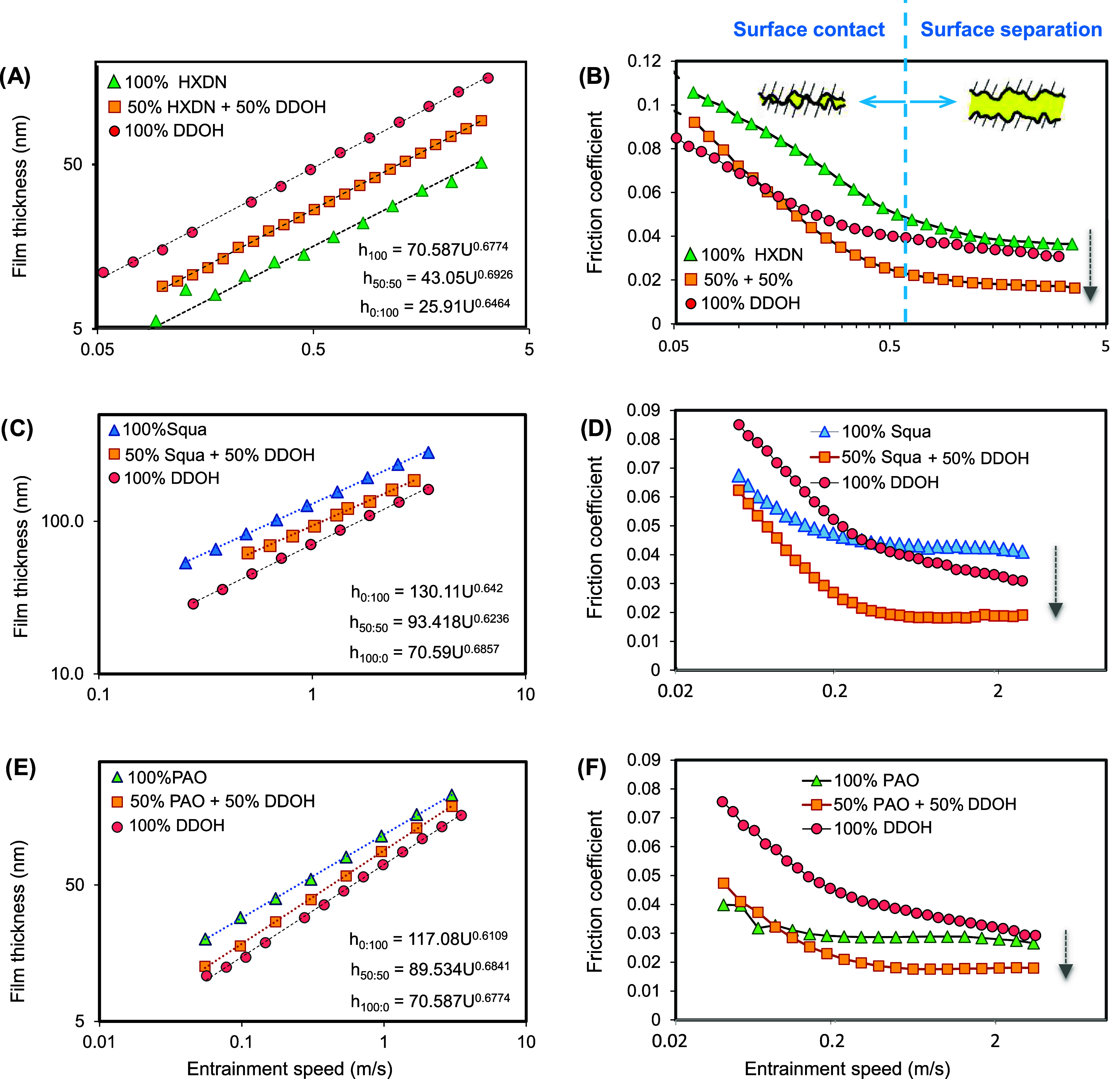
Friction coefficient and central film thickness vs entrainment speed for (A, B) neat hexadecane, neat dodecanol, and a 50:50 blend of the two; (C, D) neat squalane, neat dodecanol, and a 50:50 blend of the two; and (E, F) neat PAO, neat dodecanol, and a 50:50 blend of the two. In all cases, the applied load is 20 N, and the lubricant temperature is 40 °C.

Figure 2A shows the variation in EHL central film thickness with speed for pure hexadecane, pure dodecanol, and a 50:50 blend of the two, measured by ultrathin-film interferometry (UTFI). This is classical EHL behavior, in which the log of the film thickness increases linearly with the log of entrainment speed (according to the power law relationship in eq 1) and increases with lubricant low-pressure viscosity in order η_hexadecane_ < η_50:50_ < η_dodecanol_ (also predicted by eq 1). The Stribeck curve^57^ in Figure 2B shows the variation in friction with entrainment speed (the latter being a proxy for film thickness according to eq 1), measured by MTM for the same lubricants as in Figure 1A. At low speeds (*i.e*., low film thickness), the pressure generated by the entrained lubricant is insufficient to support the applied load and metal–metal contact occurs, giving high friction. As speed increases, friction decreases as the hydrodynamic pressure generated begins to push the surfaces apart, and asperity contact is replaced with increasingly large regions of the fluid lubricant. Once the surfaces are fully separated, the curve flattens as friction arises solely from shearing of the viscous lubricant film. The ranking of the friction values in Figure 2B is highly anomalous—rather than increasing with low-pressure viscosity, the order is μ_50:50_ < μ _hexadecane_ < μ _dodecanol_. Thus, adding dodecanol to hexadecane increases the low-pressure viscosity and hence boosts film thickness and simultaneously decreases the EHL viscous friction. These benefits are not usually concurrent since a difference in viscosity between two blend components should, according to eqs 1 and 3, have opposite effects on film thickness and viscous friction/traction. Since EHL film thickness is determined by the conditions at the low-pressure contact inlet, while EHL friction results from shearing of the lubricant in the high-pressure contact zone, the addition of dodecanol must increase the effective viscosity of hexadecane at lower pressures but reducing it at higher pressures (*i.e*., according to eq 2, η_blend_(*p*_inlet_) > η_hexadecane_(*p*_inlet_) and η_blend_(*p*_contact-zone_) < η_hexadecane_(*p*_contact-zone_)).

### Effect of Base Oil

The 50:50 mixtures of dodecanol and other hydrocarbon lubricants were tested (Figure 2C–F). As these Stribeck curves show, the effect of mixing this *n*-alcohol with these different lubricants causes a reduction in EHL friction (at high entrainment speeds), irrespective of whether the oil has (i) multiple short branches as for squalane (Figure 2C) or (ii) fewer longer branches such as for poly-α-olefin (PAO) (Figure 2E). These results are indicative of many other tests we have run (including fully formulated commercial lubricants, see Figure S3 in the Supporting Information), which show that the friction-reducing behavior of alkanols occurs independently of film thickness and irrespective of the structure and formulation of the lubricant with which it is blended. Importantly, the boundary friction (at low entrainment speeds) of the 50:50 blends is not adversely affected compared to that of the pure base oil. The boundary friction can be more easily reduced with other additives, namely, friction modifiers, which the alkanol component is not expected to interfere with. This is because, unlike friction modifiers,^7^ alkanols are not very surface-active and their EHL friction reduction mechanism acts in the bulk oil^37^ rather than at the surface. A specific investigation of this potential synergy with friction modifiers^58^ will be the topic of future research.

In addition to causing this anomalous friction reduction, blending an alkanol into an oil also affects the lubricant viscosity. In other words, unless the alkanol has the same viscosity and pressure viscosity coefficient as the oil, the resulting mixture will have a different film thickness than the oil, irrespective of the anomalous hydrogen-bonding behavior. For instance, if the alkanol’s viscosity and pressure viscosity coefficient are higher than the oil, then the film thickness will increase (which is the case for *n*-hexadecane, as shown in Figure 2A). Conversely, as squalane and PAO have a higher viscosity than *n*-dodecanol, they produce thicker EHL films (Figure 2C,E). In practice, the viscosity of the blend can be easily adjusted by varying the viscosity of the base oil to achieve the desired viscosity grade.^59^ This enables anomalous friction reduction to be achieved independently of lubricant viscosity and film thickness performance and is an approach that can be implemented in practice to yield low friction while also meeting a specific viscosity requirement. This approach is demonstrated for the fully formulated oil results in Figure S3 in the Supporting Information, where the base oil components in the blend were adjusted so its viscosity equaled that of the unblended oil.

### Effect of Pressure

**Figure 3:**
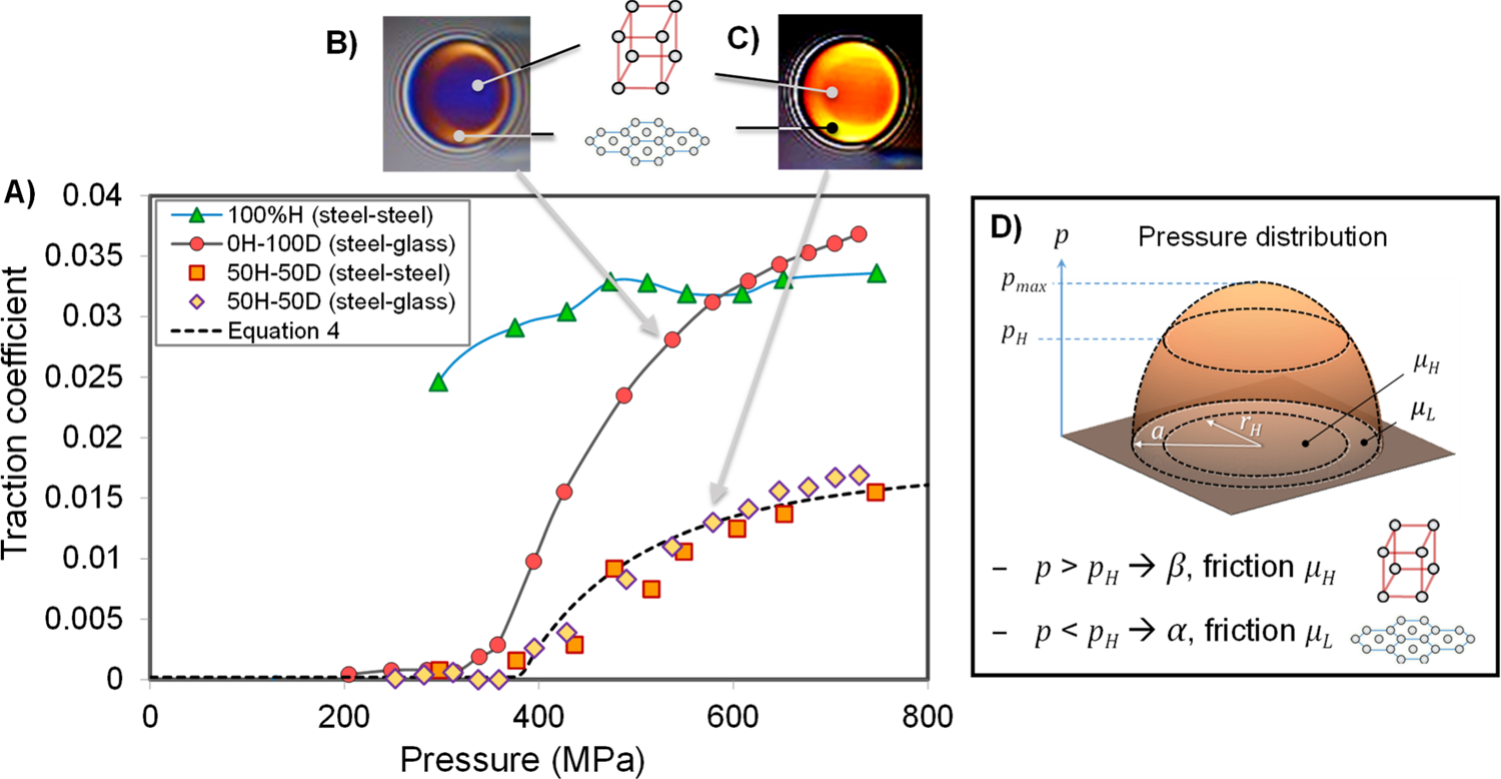
Effect of pressure on friction. (A) EHL friction coefficient vs maximum Hertz contact pressure for hexadecane, dodecanol, and a 50:50 blend of the two. Optical interferometry images of contact lubricated by (B) neat dodecanol and (C) 50:50 dodecanol–hexadecane blend. The lubricant temperature is 40 °C, the slide-roll ratio is 50%, and the entrainment speed is 2 ms^–1^. (D) Schematic of EHL pressure and friction coefficient distribution for the phase-changing lubricant.

Figure 3 shows the friction response to changes in maximum Hertz contact pressure (achieved by varying the applied normal load) for pure dodecanol, pure hexadecane, and a 50:50 blend of the two. The response of pure dodecanol shows a flat, superlubricious region at low pressures, followed by a discontinuity before increasing asymptotically toward a constant value. This behavior has been studied in detail for pure dodecanol^37^ and has been attributed to a pressure-induced phase change from the hexagonal close-packed arrangement (whose graphene-like layers are easily sheared, thus yielding superlubricity) to an orthorhombic crystal region (that is rigid and therefore gives high friction), above the phase transformation pressure, which grows to cover a larger proportion of the contact as the applied load increases.

The friction response of the dodecanol–hexadecane blend to increasing pressure is like that of pure dodecanol (i.e., an initial superlubricious valley followed by a discontinuity, before increasing asymptotically toward a constant value). Furthermore, the friction curves for the blend obtained from tests using different disc specimen material combinations collapse onto a single master curve when plotted against maximum Hertz pressure, suggesting that bulk, pressure-induced phase changes occur irrespective of surface chemistry. Also, the film thickness map obtained by EHL-SLIM for the hexadecane–dodecanol blend (Figure 3C) exhibits a circular discontinuity reminiscent of the pure dodecanol case (Figure 3B). This may be due to either an abrupt increase in film thickness (i.e., dimple formation) in response to a reduced average flow speed due to solidification^37,53^ or a change in the refractive index. Both explanations suggest a pressure-induced phase change occurring above a threshold pressure within the contact zone.

The hypothesis that the anomalous variation in friction with applied load is due to a phase change occurring at a certain pressure threshold can be tested by fitting eq 4 to the data. The results in Figure 3A show that there is a good agreement (*R*^2^ = 0.952) between the experimental friction coefficient vs load graph for the blend and eq 4, with the only adjustable constants being *p*_H_ = 0.38 GPa, μ_L_ = 0.0002, and μ_H_ = 0.018. This further confirms that a phase change occurs at a specific pressure.

The above evidence strongly suggests that the same pressure-induced phase changes (from low-friction hexagonal close-packed α-phase to high-friction orthorhombic β-phase) occur for alcohol molecules in the blend as they do for the neat alkanol. The question remains as to why these phase changes result in such low friction for the blend (particularly at high pressures where the neat dodecanol exhibits high friction). Notably, the suggested transformation from hexagonal close-packed α-phase to orthorhombic β-phase occurs at a slightly higher pressure for the hexadecane–dodecanol blend than for pure dodecanol (0.38 MPa vs 0.35 MPa), suggesting that the presence of hexadecane inhibits the transformation.

Figure 3 also shows that, at low pressures, the hexadecane–dodecanol blend exhibits superlubricity (with all CoF values <0.0004) due to the hexagonal close-packed dodecanol α-phase. Given the similarities in their melting points, it is likely that both dodecanol (mp = 24 °C) and hexadecane (mp = 18 °C) molecules have solidified here and that these solid phases will have low solubility.^60^ However, it should also be noted that a 50:50 wt mixture of dodecanol and PAO produces a friction–load plot with the same shape as that in Figure 3, which suggests superlubricity and the transformation between phases are not specific to the linear chained hexadecane (see Figures S1 and S2 in the Supporting Information). Branched alkanes PAO and squalane (mp = −38 °C) have much lower melting points than linear dodecanol and hexadecane and will form glasses rather than crystalline solids inside high-pressure EHL contacts.^61^

### Effect of Shear Rate

**Figure 4:**
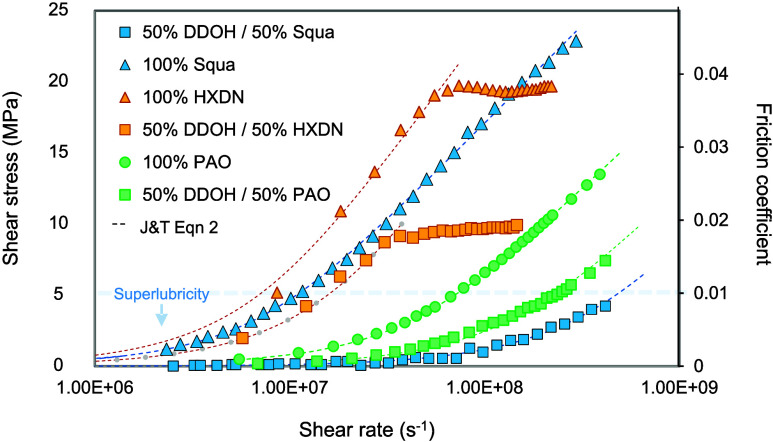
Effect of shear rate: shear stress vs strain rate for contacts lubricated by straight and branched hydrocarbon lubricants and mixtures with dodecanol. The load is 20 N (mean Hertz pressure = 548 MPa), and the entrainment speed is 2 ms^–1^. The Johnson and Tevaarwerk equation (eq 2) is fitted to each graph, with the resulting parameters given in Table 1.

Figure 4 plots the results from an MTM traction curve test in the form of average contact shear stress, *τ*, vs strain rate, *γ̇*. This is obtained from a test in which the slide-roll ratio (SRR) is varied and converted to strain rate by γ̇ = *SRR* × *U*/*h* (since γ̇ = Δ*U*/*h* and SRR = Δ*U*/(*U̅*), where Δ*U* = *u*_1_ – *u*_2_ and *U̅* = 0.5(*u*_1_ + *u*_2_)), while the measured coefficient of friction, *μ*, is converted to shear stress by τ = μ*p*. The EHL film thickness, *h* ≈ *kU̅*^0.67^, is kept constant by maintaining an entrainment speed, *U̅*, of 2 ms^–1^. This is fast enough (according to eq 1) to ensure that the surface separation is large compared to the composite surface roughness, and the contact operates in the full-film EHL regime, where friction losses arise solely from lubricant shear rather than surface contact.

The curves for pure squalane and the PAO in Figure 4 agree with eq 2, showing a Newtonian dependence of shear stress on strain rate at low shear rates and an approximately linear dependence on log γ̇ at higher shear rates. Without thermal correction, it is expected that these curves flatten at higher shear rates,^62^ (as the final three data points for the neat squalen indicate). The pure hexadecane curves show signs of reaching a limiting shear stress at high shear rates, possibly resulting from slip^63^ due to solidification, thanks to its high melting point.

The 50:50 blends of these hydrocarbon lubricants with dodecanol again show a significant reduction in friction compared to that of the neat fluids across all shear rates (Figure 4). This suggests that the phase changes in the alcohol do not depend on shear stress or strain rate^64^ in a discontinuous way as they do on pressure (Figure 3). Instead, each blend curve follows a similar shape to that of the oil with the alkanol it is blended with, which may suggest that the dissipation within the blends is associated with the shearing of the hydrocarbon portion rather than the alcohol. This was analyzed by fitting the Johnson and Tevaarwerk equation (eq 2) to the data in Figure 6 to estimate the effect of alkanol dilution on the hydrocarbon’s Eyring stress, τ_e_, and mean low shear rate viscosity, η_*p*_ (here, the predicted friction coefficient was obtained from eq 2 by dividing the average shear stress by the mean Hertz contact pressure). For the hexadecane curve, eq 2 is fitted only up to the limiting shear stress plateau. It became apparent that τ_e_ was largely unaffected by dilution, while η_*p*_ changed significantly. To test this, eq 2 was fitted to the data, with τ_e_ set to the same value for the neat oil and the blend, while η_*p*_ was unconstrained. The resulting fitted curves (detailed in Table 1) agree with the experimental data in Figure 4, all with *R*^2^ > 0.9, showing that, when the tested lubricants are diluted with dodecanol, the mean low shear rate viscosity, η*_p_*, reduces by ∼56, 69, and 98% for hexadecane, PAO, and squalane, respectively. This suggests that the anomalously low friction of the dodecanol blends may be caused by a reduction in high-pressure viscosity rather than an increase in shear thinning or liquid slip. However, further rheological testing is required to confirm this. This viscosity reduction correlates with the degree of branching, DB (DB = *R*/*R*_max_, where *R* is the number of branches and *R*_max_ is the length of the longest chain), of the hydrocarbon oil molecules, as presented in Table 1, which suggests a mismatch in structure between the two sheared components is responsible.

**Table 1:** Approximate Lubricant Parameters Obtained by Fitting the Johnson and Tevaarwerk Equation (eq 2) to Data in Figure 6

test lubricant	degree of branching, DB	Eyring stress, τ_e_ [MPa]	effective viscosity, η_p_ [Pa s]	*R*^2^ of the fitted curve	viscosity reduction [%]
100% hexadecane	0.00	8.0	0.77	0.987	
50% hexadecane/50% 1-dodecanol	0.00/0.00	8.0	0.34	0.941	56
100% PAO4	0.10	5.64	0.081	1.000	
50% PAO4/50% 1-dodecanol	0.10/0.00	5.64	0.025	0.987	69
100% squalane	0.25	5.99	0.51	1.000	
50% squalane/50% 1-dodecanol	0.25/0.00	5.99	0.012	0.986	98

Fitting the Johnson and Tevaarwerk equation is used here to quantify the similarity in shape of the shear stress vs strain rate graphs (using just two adjustable constants) and assess the relative contributions of shear thinning vs bulk viscosity change to the observed EHL friction reductions. This is not intended as a way of determining bulk lubricant properties—for more accurate high-pressure viscosity measurements of neat PAO and Squalane, see Bair’s measurements.^65^

At lower shear rates, the squalane–dodecanol blend gives extremely low shear stress, corresponding to superlubricious friction coefficient values <0.01 for all measured slide-roll ratios. Here, the mean Hertz contact pressure for this test is 548 MPa, which is high enough, according to Figure 3, to cause the “high-friction” orthorhombic β-phase to form for pure dodecanol. The shear stress/friction reduction of the squalane blend becomes less pronounced at higher speeds due to the shearing of the squalane fraction.

### Effect of Concentration

**Figure 5:**
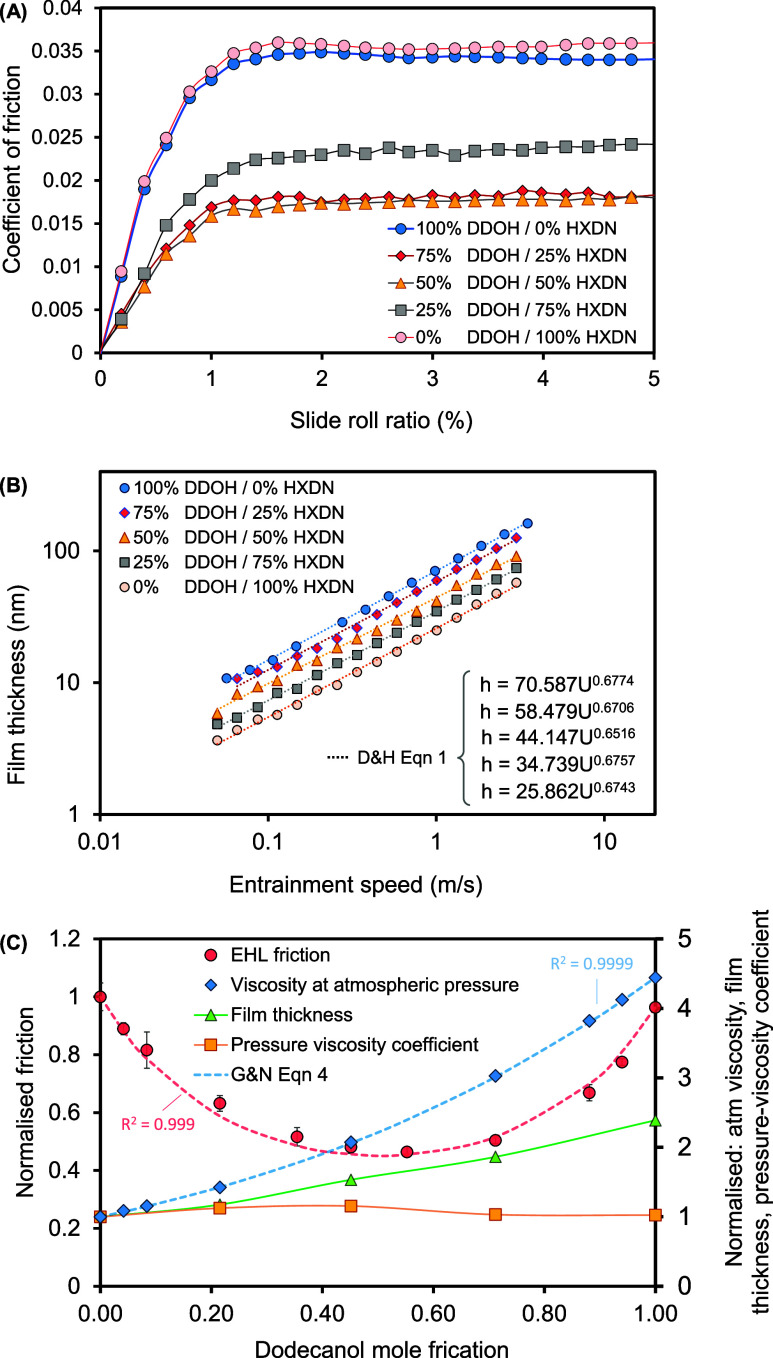
Effect of concentration. (A) Coefficient of friction vs slide-roll ratio (SRR); (B) central film thickness vs entrainment speed for different concentrations of hexadecane and dodecanol; and (C) percentage reduction in friction (μ_blend_/μ_oil_), variation in viscosity measured at atmospheric pressure, measured film thickness and pressure viscosity coefficient calculated from film thickness data in (B) vs mole fraction. Tests were performed at 40 °C temperature, 20 N load (mean Hertz pressure = 548 MPa), and 2 ms^–1^ entrainment speed throughout.

The effect of dodecanol concentration on EHL friction and film thickness is shown in Figure 5. Here, film thickness increases monotonically with increasing dodecanol concentration (classical EHL behavior), while friction shows an anomalous trend that decreases and then increases. To summarize the friction-reducing effect of dodecanol concentration, results are normalized by dividing each point on each traction curve by the corresponding point for pure hexadecane. This produced the result shown in Figure 5C. The minimum friction in Figure 5C occurs at a mole fraction of approximately 50:50 (considering that the molar masses of dodecanol and hexadecane are 186.34 and 226.445 g/mol, respectively). This may suggest that the anomalous reduction in EHL friction does not occur due to wall slip, resulting from a surface alcohol film (as only a low concentration, e.g., <1%, of additive molecules is required to form a complete surface film as predicted by refs (7,66)), and instead points to a bulk change in rheology occurring within the contact.

The EHL film thickness and ambient pressure viscosity of the blends are plotted in Figure 5C, both of which increase monotonically as the more viscous dodecanol is added at increasing concentrations to the hexadecane. Figure 5C also shows the pressure viscosity coefficient, α-value, predicted by fitting eq 1 to film thickness data (according to ref (67)). The monotonic variation of α-value with concentration again suggests that the apparent negative excess viscosity occurs only in the central, load-supporting region of contact and is independent of the film thickness, which is determined at the contact inlet where pressures are below ∼70 MPa.^62^

The Grunberg and Nissan formula^55^ (eq 4) was fitted to the EHL friction and viscosity curves in Figure 5c, yielding *d*′ interaction parameter values of −3.1 and 0.1, respectively. This suggests that the interactions between dodecanol and hexadecane molecules change from slightly attractive to very weakly attractive because of the pressure applied to the lubricant within the contact.^56^ Note: other, more detailed mixing equations (*e.g*., Redlich–Kisler,^68^ McAllister,^69^ UNIFAC^70^) could also be used here.

### Ex Situ Measurements

**Figure 6:**
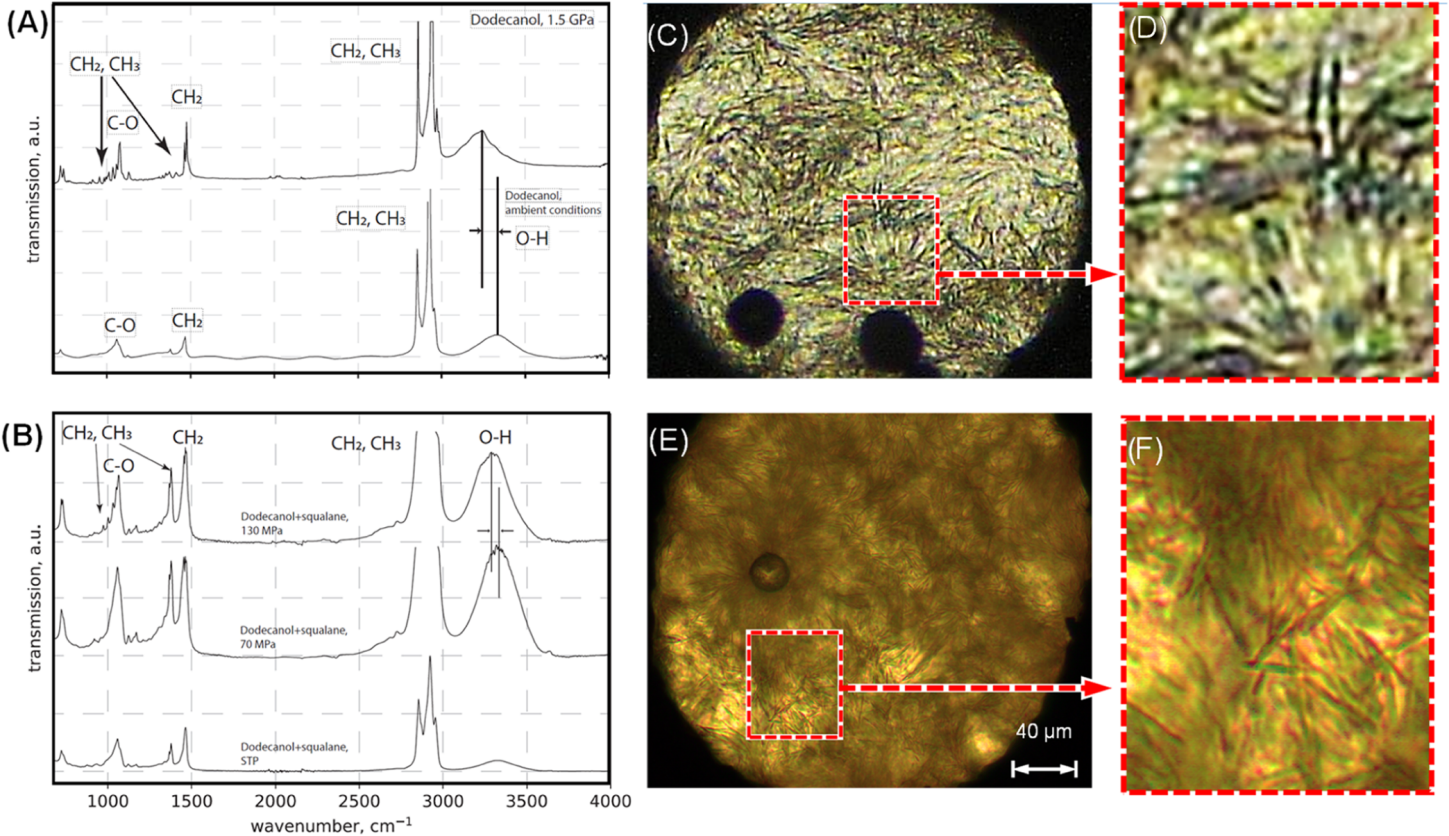
Diamond anvil cell data. FTIR spectra for pure dodecanol (A) at low (bottom) and high (top) pressures (measured at B22, Diamond Light Source)^37^ and (B) 50:50 dodecanol–squalane blend (measured at Natural History Museum, London). Vibrational assignments are based on ref (76). (D–F) Polarized light microscopy images of the DAC chamber with (C, D) 50:50 blend of squalane:dodecanol by wt. at 25 °C and 130 MPa and (E, F) neat 1-dodecanol at 25 °C and 1.5 GPa.

Ex situ measurements were conducted to probe the mechanisms responsible for the anomalously low friction of the dodecanol blends. First, a diamond anvil cell (DAC) was used to obtain FTIR spectra of a 50:50 squalane–dodecanol blend at low (70 MPa) and high (130 MPa) pressures, as shown in Figure 6B. For comparison, the FTIR spectra of neat dodecanol are shown in Figure 6A.

Comparing the high- and low-pressure spectra of pure dodecanol in Figure 6A shows that the CH_2_ and CH_3_ peaks at 1000 and 1400 cm^–1^ have intensified (indicating pressure-induced solidification^71^) and the OH peak at ∼3200 cm^–1^ has shifted and intensified. The latter indicates the formation of a network of hydrogen-bonded hydroxyl groups, which has previously been associated with solidification by cooling in *n*-octanol^72^ and by pressurization in formic acid.^71^ Furthermore, high-pressure dodecanol shows a series of sharp peaks at around 1370–1250 and 950–1030 cm^–1^. As noted by Pocheć et al.,^73^ these are due to wagging deformations of the chain and stretching of the C–C bonds within the chain, respectively.^74,75^ Importantly, these pressure-induced CH_2_, CH_3_ and OH changes can also be observed in the spectra for the squalane–dodecanol sample (Figure 6B), suggesting that a pressure-induced hydrogen-bonded dodecanol network also forms here. Visual observations within the DAC confirm that this phase change was reversible on depressurization. This observation agrees with interference images in Figure 3B,C, which show that the anomalous central region ends before the lubricant exits the contact, presumably when the pressure falls below the solid–liquid transformation pressure.

Optical microscopy was used to view the DAC containing the 50:50 dodecanol–squalane blend pressurized to 150 MPa at 25 °C (conditions known to cause neat dodecanol to solidify into the orthorhombic phase.^37^) Here, the blend shows the formation of needlelike crystals approximately <1 μm wide and up to 200 μm long (Figure 6E,F), which are reminiscent of those formed by neat dodecanol (Figure 6B,C^37^). These randomly aligned, needlelike crystals observed in the DAC on gradual, isostatic loading are unlikely to have the same size, shape, and orientation as the pressure-induced phase changes responsible for the anomalous friction in the simulated bearing contact. This is because, in the DAC, loading occurs over ∼1 s and is approximately isostatic, whereas in the bearing contact, the pressure increase occurs over ∼50 μs and is subject to significant shear stresses, which will affect crystal orientation (so that their weakest axis is perpendicular to the unique stress axis^77^). Nevertheless, these DAC images further support the hypothesis that the pressure threshold above which anomalously low friction occurs in the bearing contact corresponds to a phase change of the dodecanol, which results in phase separation of the blend. Moreover, this correspondence between stationary DAC results (Figure 6) and sliding EHL rig results (Figure 3) is congruent with the observation that the anomalous friction reduction is largely independent of the applied shear rate (Figure 4).

**Figure 7:**
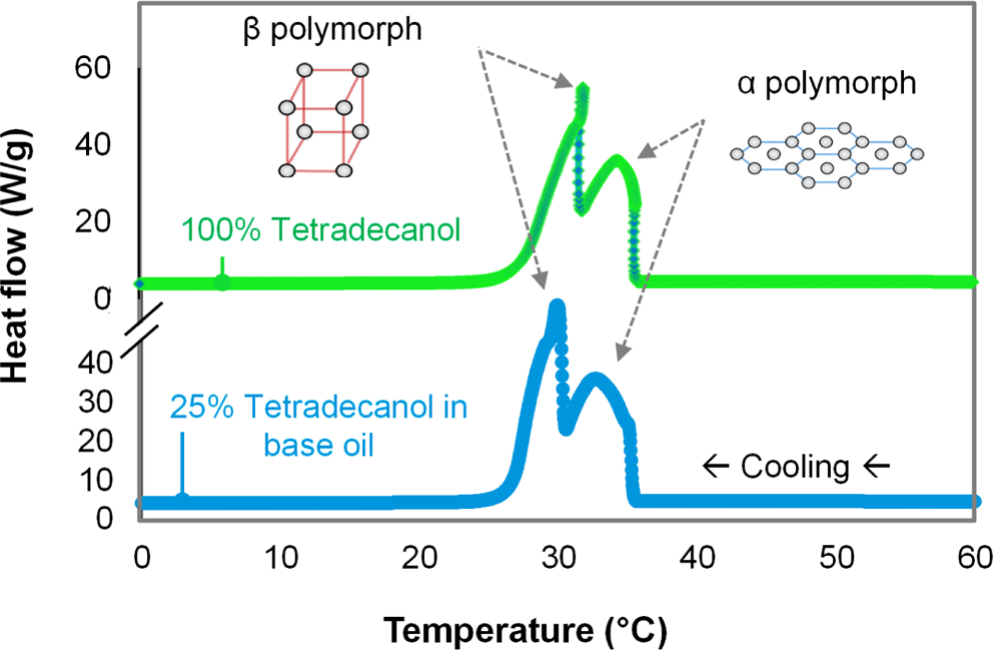
Differential scanning calorimetry for neat tetradecanol and a mixture of tetradecnaol (wt 25%) and PAO (wt. 75%). 1-Tetradecanol was used for these DSC experiments since this alkanol shows clearer exotherms/endotherms than 1-dodecanol,^37^ while both exhibit the same anomalous friction behavior reported here.

DSC data for a neat alkanol and an alkanol–PAO blend are shown in Figure 7. The two exotherm peaks for the neat sample correspond to phase transformations during cooling: first, from a disordered liquid to a hexagonal close-packed α-phase and subsequently to an orthorhombic β-phase.^51,52^ Importantly, this series of phase transformations that occurs for neat alkanols due to cooling at atmospheric pressure has been shown to occur in EHL contacts due to increased pressure at constant temperature and is the cause of the observed friction transitions (Figure 3)^37^ (the converse effects of pressure and temperature on crystallization are well known^78^). Comparing the exotherms for the neat alkanol and the alkanol–PAO blend in Figure 7 suggests that these same alkanol phase transformations occur even when diluted. The endotherms produced by cooling the mixture occur at a lower temperature than the neat alkanol, probably because the branched PAO lubricant hinders crystallization. Note that *n*-tetradecanol, C_14_H_30_O, was used for DSC measurements instead of *n*-dodecanol, C_12_H_26_O, since both demonstrate the same anomalous friction behavior (see Figure S3 in the Supporting Information), while the former exhibits clearer DSC peaks. This is because, compared to the rate of pressure increase that a sample experiences inside an EHL contact (0.1 MPa to 0.5 GPa in ∼50 μs), the slow DSC cooling rate of 40 °C min^–1^ limits the formation of the α-phase. This is analogous to kinetic control in polymorph selection of other molecular solids.^79^

## Discussion

Mixtures of dodecanol and different hydrocarbon lubricants are shown here to produce anomalously low full-film elastohydrodynamic friction (Figures 2–5) lower than either fluid individually—which appears to contradict conventional theory (eq 3). Furthermore, under some conditions, friction is lower than the superlubricity threshold. Beneficially, the thickness of the entrained film for these blends still obeys the established EHL theory (eq 2) and thus depends predominantly on low-pressure viscosity, which varies monotonically depending on the proportion of individual fluid components (Figure 5). This discrepancy must be due to the blends having a lower effective viscosity in the high-pressure contact zone where friction arises, compared to in the low-pressure inlet where the film thickness is determined. This decoupling of high- and low-pressure behavior is exemplified in Figure 5, where the nominal pressure viscosity coefficient (calculated from the film thickness) increases monotonically with dodecanol concentration, while the EHL friction shows a minimum, which is 50% lower than that of either of the neat constituents. We suggest the low effective viscosity is caused by pressure-induced phase changes in the dodecanol molecules within the blend, specifically a change from a disordered liquid to a hexagonal close-packed α-phase to an orthorhombic β-phase with increasing pressure. This hypothesis is supported by (i) the discontinuous pressure dependence of the blends following the same characteristic shape as that for pure dodecanol (which is known to form these polymorphs^80^ depending on the pressure distribution within the contact^37^) with nearly the same phase change pressures (Figure 3A), (ii) in-contact interference images of the blend showing an approximately circular colored region corresponding to a pressure contour within the approximately Hertz pressure profile (Figure 3C), (iii) FTIR spectra from a squalane–hexadecane blend pressurized within a DAC showing key features that suggest a ordered hydrogen-bonded network, which are reminiscent of those exhibited by neat dodecanol (Figure 6A,B), (iv) similar crystal morphology for pressurized dodecanol–lubricant mixtures and pure dodecanol (Figure 6C–F), and (v) DSC of the alkanol–lubricant mixtures showing the same phase changes as pure alkanols (which themselves are known to result from disordered liquid → α-phase → β-phase transitions^51,52^). There is a question of why dodecanol forms these two polymorphs. This may be because the hexagonal α-phase represents a molecular packing arrangement (or mesophase^81^) that locally minimizes enthalpy through translational ordering while frustrating the formation of the globally minimal free-energy crystalline state (β-phase).^82,83^ Dodecanol in this α-phase may therefore be considered supercooled/supersaturated—a thermodynamically unstable state that remains kinetically stable due to the lack of nucleation or crystallization triggers.^79^

The superlubricity region observed for the hexadecane–dodecanol blend below ∼400 MPa (Figure 3) likely occurs due to interfacial sliding of hexagonal α-phase arrangements of dodecanol known to be present at these pressures^37^ between heterojunctions,^84^ facilitated by stress-orientation crystallization.^77^ Beneficially, this occurs without a graphene treatment applied to the specimen surface, as recent demonstrations under boundary lubrication conditions have required.^84^ This may be due to lower shear stress under EHL conditions, as the high-pressure region is distributed over the Hertz contact area rather than localized at asperity contacts. More generally, compared to most demonstrations of synthetic superlubricity,^14^ this is remarkably close to commercial lubricants in an actual bearing interface.

The presence of the more rigid orthorhombic β-phase within the blend also gives anomalously low friction. For instance, the dodecanol/hydrocarbon mixture exhibits friction lower than that of either of its constituents individually, even when the pressure is sufficiently high to produce solid orthorhombic, high-friction β-phase crystals. Specifically, the low-friction blend results in Figures 2, 4, and 5 were all obtained at pressures above the α → β transition shown in Figure 3. This friction reduction with increased heterogeneity may be an example of negative excess viscosity—i.e., a binary mixture with a viscosity lower than either of its neat constituents.^68,69,85,86^ In fact, the Grunberg and Nissan formula^55^ typically used to predict excess viscosity fits the friction data in Figure 5C well (*R*^2^ = 0.999). Many such mixtures, albeit measured at ambient pressure, show viscosity vs concentration curves identical to the friction concentration data for the dodecanol–hexadecane blend in Figure 5C. For example, Mahajan et al. reported negative viscosity deviations for tetradecane/octan-2-ol mixtures, attributed to dilution disrupting the self-associated alcohol molecules and more weak interactions between unlike molecules.^87^ This type of viscosity-modifying OH···OH self-association effect is likely to occur as our blends enter an elastohydrodynamic contact and produce friction, given that dodecanol becomes crystallizable under these conditions. Moreover, Papaioannou et al. showed that the excess viscosity of alkanol/*n*-butylamine can be controlled by applying pressure.^88^ This pressure-induced negative excess viscosity hypothesis is further supported by the ranking of the high-pressure viscosity reductions for the three base oils in Table 1 (hexadecane 50%, PAO 69%, and squalane 97%), which corresponds to the degree of branching in these molecules. The most branched/straight blend, squalane/dodecanol, is sufficiently heterogeneous to cause superlubricity across all shear rates tested, even under high-friction β-phase conditions.

Other hypotheses requiring further investigation include dodecanol affecting the viscoelastic response of the blend^89^ or alternatively acting as a plasticizer and reducing viscosity by delaying the glass transition to higher pressure (as fatty acids and fatty acid esters may do^90^).

These friction-controlling mechanisms may be utilized in practice by including hydrogen-bonding additives, such as n-alcohols, in commercial lubricants to reduce energy consumption across a range of machines in which EHL contacts are present. This endeavor is facilitated by (1) stability of the mechanism over a range of pressures (Figure 3), strain rates (Figure 4), and entrainment speeds (including high speeds where power dissipation is the greatest, Figures 2 and 5), (2) effectiveness of the mechanism across different base oil structures (Figure 2), (3) reversible nature of hydrogen bonding, meaning that these additives are not depleted during use (Figure 3C), (4) exploitable synergies with the base oil structure (e.g., increased branching promotes superlubricity, Table 1), (5) relatively low cost of *n*-alcohols, which is comparable to a high-quality base oil, thanks to mass production through a range of methods^91^ (many of which are from biomass such as the hydrolysis of triglycerides and fatty acids^91^), and (6) low toxicity of *n*-alcohols (they are used in many cosmetic products^91^). Before implementation, research is needed into permissible alkanol concentrations that meet lubricant specifications, such as oxidation stability, viscosity index, and interactions with other additives. Fortunately, a myriad of molecular structures of alcohols may be explored to enhance performance (or maintain performance at reduced alkanol concentration). Furthermore, the superlubricity results shown here suggest that lower, less intrusive concentrations can yield significant friction deductions. This raises the possibility of alkanol ‘traction modifier’ additives as a complement to conventional surface-active friction modifier additives, which are worth >1 billion USD per year,^92^ despite only being effective under nonoptimal low-speed surface contact conditions. Traction modifier additives would also complement the currently constrained trend toward lower-viscosity lubricants.^45^

## Conclusions

Mixing an *n*-alcohol with a hydrocarbon oil reduces the EHL friction without adversely affecting film thickness. This is due to the pressure-induced formation of the *n*-alcohol’s solid α/hexagonal and β/orthorhombic polymorphs, coupled with the fact that film thickness is determined by conditions in the low-pressure inlet, while friction is determined within the loaded zone. Both phase changes reversibly reduce the lubricant’s local bulk viscosity without depleting the alcohol. This mechanism is robust over a range of base oil molecular structures, entrainment speeds, applied pressures, and shear rates. Furthermore, superlubricity can be achieved using either (1) pressure–temperature conditions that favor dodecanol’s laminar α-polymorph or (2) a branched oil such as squalane. Compared to most demonstrations of synthetic superlubricity,^14^ this is remarkably close to actual lubricants in an actual bearing interface. Moreover, *n*-alcohols can be sustainably produced from renewable sources and are relatively low-cost and nontoxic. With appropriate modeling, optimization, and no-harm testing, these blends may provide a genuine energy-saving solution in certain commercial lubricants.

## Materials and Methods

### Materials

1-Dodecanol and 1-tetradecanol (>98%) were purchased from Sigma-Aldrich (CAS Nos. 112-53-8 & 112072-1). 1-Dodecanol, the longest n-alcohol that is a liquid at room temperature, is low-cost and can be sourced from renewable resources.^93^ The model hydrocarbon lubricants with which 1-dodecanol was blended were hexadecane (linear) and squalane (branched), both purchased from Sigma-Aldrich (CAS Nos. 544-76-3 and 111-01-3, respectively). The widely used synthetic base oil PAO4 (4cSt at 100 °C) was also blended with dodecanol and tested. A fully formulated oil containing a commercial additive package was blended with tetradecanol and tested (see Supporting Information).

## Methods

The test lubricants’ EHL friction and EHL film thickness performance in simulated ball bearing contacts were measured, and then, the underlying mechanisms were probed in a diamond anvil cell (DAC).

**Figure 8:**
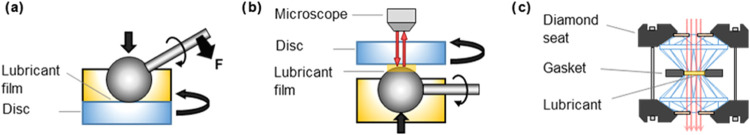
Schematic diagrams of the test apparatus. (A) Mini-traction machine for EHL friction measurement, (B) ultrathin-film interferometry rig for EHL film thickness measurement, and (C) diamond anvil cell for ex situ high-pressure measurements, from ref (37).

EHL friction coefficient measurements were made using a mini-traction machine (MTM; PCS Instruments), shown schematically in Figure 8A. The MTM simulates a bearing or gear contact by loading together a ball and disc and driving each with independent motors to facilitate different combinations of rolling and sliding speeds.^94^ The resulting shear rate of the lubricant is approximated by the slide-to-roll ratio, SRR = Δ*U*/(*U̅*), where Δ*U* = *u*_1_ – *u*_2_ and *U̅* = 0.5(*u*_1_ + *u*_2_), in which *u*_1_ and *u*_2_ are the linear speeds of the ball and disc surface, respectively. A piezoelectric transducer connected to the ball shaft holder measures the friction force. The steel ball (elastic modulus *E* = 207 GPa, Poisson’s ratio *υ* = 0.3^95^) has a diameter of 19.05 mm and was in a temperature-controlled lubricant bath (accurate to ±1 °C) to ensure that a fluid film is entrained into the contact. Both steel and glass (*E* = 81 GPa, *υ* = 0.208^96^) discs were used to achieve different contact pressures.

Elastohydrodynamic (EHL) central film thickness was measured by using an EHL film thickness test rig (PCS Instruments). This loads a glass disc against a 19.05 mm diameter AISI 52100 steel ball, which is partially submerged in a lubricant that becomes entrained between the specimens during rotation (Figure 8B). The disc is coated with chromium and silica layers of approximately 20 and 500 nm thicknesses, respectively. This enables central film thickness to be obtained by UTFI, whereby a spectrometer disperses light reflected from the contact into component wavelengths to identify those that interfere.^97^ This requires knowledge of the lubricant sample’s refractive index, which is measured beforehand and assumed to remain constant during the test. Any effect of alkanol phase transitions on the refractive index and hence measured film thickness has been shown in refs (37,53) to be slight (∼5%).

Diamond anvil cell (DAC) measurements were made using the setup shown in Figure 8C. Here, a pair of type II diamonds with 500 μm culets was clamped together over a 316 stainless steel gasket (250 μm) preindented to 100 μm with a ∼200 μm diameter hole. The liquid sample was loaded with a small (50–80 μm diameter) ruby sphere (used for pressure determination). The entire assembly was placed in an iN10mx FTIR microscope (Thermo Fisher) to collect FTIR spectra and under a petrographic microscope (Zeiss) to view crystal formation.^98^

Differential scanning calorimetry (DSC) was used to detect phase transformations in dodecanol by measuring the amount of heat required to increase/decrease the sample temperature at a constant rate. Positive and negative changes in the heat input indicate exothermic and endothermic phase transformations.

## Supporting Information Available

Blends of dodecanol with squalane and polyalphaolefin produce similar friction vs pressure behavior to blends with hexadecane (Figure 3) and alkanol-induced friction reduction can occur in a fully formulated oil, as demonstrated when blended with the C14 alkanol, tetradecanol (PDF)

## References

[ref1] Holmberg K.; Erdemir A. Influence of Tribology on Global Energy Consumption, Costs and Emissions. Friction 2017, 5, 263–284. 10.1007/s40544-017-0183-5.

[ref2] Taylor R. I. Tribology and Energy Efficiency: From Molecules to Lubricated Contacts to Complete Machines. Faraday Discuss. 2012, 156, 361 10.1039/c2fd00122e. 23285639

[ref3] Spikes H. A. A Thermodynamic Approach to Viscosity. Tribiol. Trans. 1990, 33 (1), 140–148. 10.1080/10402009008981940.

[ref4] Korcek S.; Sorab J.; Johnson M. D.; Jensen R. K. Automotive Lubricants for the next Millennium. Ind. Lubr. Tribol. 2000, 52 (5), 209–220. 10.1108/00368790010373175.

[ref5] Macián V.; Tormos B.; Bermúdez V.; Ramírez L. Assessment of the Effect of Low Viscosity Oils Usage on a Light Duty Diesel Engine Fuel Consumption in Stationary and Transient Conditions. Tribol. Int. 2014, 79, 132–139. 10.1016/j.triboint.2014.06.003.

[ref6] Lee P.; Zhmud B. Low Friction Powertrains: Current Advances in Lubricants and Coatings. Lubricants 2021, 9 (8), 74 10.3390/lubricants9080074.

[ref7] Spikes H. Friction Modifier Additives. Tribol Lett. 2015, 60, 5 10.1007/s11249-015-0589-z.

[ref8] Martini A.; Ramasamy U. S.; Len M. Review of Viscosity Modifier Lubricant Additives. Tribol. Lett. 2018, 66, 58 10.1007/s11249-018-1007-0.

[ref9] Marx N.; Ponjavic A.; Taylor R. I.; Spikes H. A. Study of Permanent Shear Thinning of VM Polymer Solutions. Tribol Lett. 2017, 65 (3), 106 10.1007/s11249-017-0888-7.

[ref10] Gabelli A.; Voskamp A. P.; Shearer S.; Ionannides E. The Service Life of Rolling Element Bearings - Stress Field and Material Response Analysis. VDI Berichte 1998, 1380, 171–195.

[ref11] Holmberg K.; Andersson P.; Erdemir A. Global Energy Consumption Due to Friction in Passenger Cars. Tribol Int. 2012, 47, 221–234. 10.1016/j.triboint.2011.11.022.

[ref12] Holmberg K.; Erdemir A. The Impact of Tribology on Energy Use and CO 2 Emission Globally and in Combustion Engine and Electric Cars. Tribol Int. 2019, 135 (March), 389–396. 10.1016/j.triboint.2019.03.024.

[ref13] Du C.; Yu T.; Zhang L.; Deng H.; Shen R.; Li X.; Feng Y.; Wang D. Macroscale Superlubricity with Ultralow Wear and Ultrashort Running-In Period (∼1 s) through Phytic Acid-Based Complex Green Liquid Lubricants. ACS Appl. Mater. Interfaces 2023, 15, 10302 10.1021/acsami.2c22402. 36755437

[ref14] Hod O.; Meyer E.; Zheng Q.; Urbakh M. Structural Superlubricity and Ultralow Friction across the Length Scales. Nature 2018, 563, 485–492. 10.1038/s41586-018-0704-z. 30464268

[ref15] Sun T.; Gao E.; Jia X.; Bian J.; Wang Z.; Ma M.; Zheng Q.; Xu Z. Robust Structural Superlubricity under Gigapascal Pressures. Nat. Commun. 2024, 15 (1), 5952 10.1038/s41467-024-49914-6. 39009569 PMC11251065

[ref16] Hirano M.; Shinjo K. Atomistic Locking and Friction. Phys. Rev. B 1990, 41, 15 10.1103/PhysRevB.41.11837. 9993633

[ref17] Sokoloff J. B. Theory of Energy Dissipation in Sliding Crystal Surfaces. Phys. Rev. B 1990, 42, 760 10.1103/PhysRevB.42.760. 9994597

[ref18] Hirano M.; Shinjo K. Superlubricity and Frictional Anisotropy. Wear 1993, 168, 121–125. 10.1016/0043-1648(93)90207-3.

[ref19] Dienwiebel M.; Verhoeven G. S.; Pradeep N.; Frenken J. W. M.; Heimberg J. A.; Zandbergen H. W. Superlubricity of Graphite. Phys. Rev. Lett. 2004, 92 (12), 126101 10.1103/PhysRevLett.92.126101. 15089689

[ref20] Martin J. M.; Donnet C.; Le Mogne T. Superlubricity of Molybdenum Disulphide. Phys. Rev. B 1999, 48 (14), 583–588. 10.1108/ilt.2000.01852aae.001. 10007345

[ref21] Feng X.; Kwon S.; Park J. Y.; Salmeron M. Superlubric Sliding of Graphene Nanoflakes on Graphene. ACS Nano 2013, 7 (2), 1718–1724. 10.1021/nn305722d. 23327483

[ref22] Liu S. W.; Wang H. P.; Xu Q.; Ma T. B.; Yu G.; Zhang C.; Geng D.; Yu Z.; Zhang S.; Wang W.; Hu Y. Z.; Wang H.; Luo J. Robust Microscale Superlubricity under High Contact Pressure Enabled by Graphene-Coated Microsphere. Nat. Commun. 2017, 8, 14029 10.1038/ncomms14029. 28195130 PMC5316838

[ref23] Müser M. H. Structural Lubricity: Role of Dimension and Symmetry. Europhys. Lett. 2004, 66 (1), 97–103. 10.1209/epl/i2003-10139-6.

[ref24] Mandelli D.; Leven I.; Hod O.; Urbakh M. Sliding Friction of Graphene/Hexagonal -Boron Nitride Heterojunctions: A Route to Robust Superlubricity. Sci. Rep 2017, 7 (1), 10851 10.1038/s41598-017-10522-8. 28883489 PMC5589749

[ref25] Li P.; Ju P.; Ji L.; Li H.; Liu X.; Chen L.; Zhou H.; Chen J. Toward Robust Macroscale Superlubricity on Engineering Steel Substrate. Adv. Mater. 2020, 32 (36), 2002039 10.1002/adma.202002039. 32715515

[ref26] Zhang Z.; Du Y.; Huang S.; Meng F.; Chen L.; Xie W.; Chang K.; Zhang C.; Lu Y.; Lin C. Te.; Li S.; Parkin I. P.; Guo D. Macroscale Superlubricity Enabled by Graphene-Coated Surfaces. Adv. Sci. 2020, 7 (4), 1903239 10.1002/advs.201903239. PMC7029642 32099768

[ref27] Chen M.; Kato K.; Adachi K. The Comparisons of Sliding Speed and Normal Load Effect on Friction Coefficients of Self-Mated Si 3 N 4 and SiC under Water Lubrication. Tribol. Int. 2002, 35, 129 10.1016/s0301-679x(01)00105-0.

[ref28] Deng M.; Zhang C.; Li J.; Ma L.; Luo J. Hydrodynamic Effect on the Superlubricity of Phosphoric Acid between Ceramic and Sapphire. Friction 2014, 2 (2), 173–181. 10.1007/s40544-014-0053-3.

[ref29] Li J.; Zhang C.; Deng M.; Luo J. Investigations of the Superlubricity of Sapphire against Ruby under Phosphoric Acid Lubrication. Friction 2014, 2 (2), 164–172. 10.1007/s40544-014-0050-6.

[ref30] Li J.; Zhang C.; Luo J. Superlubricity Behavior with Phosphoric Acid-Water Network Induced by Rubbing. Langmuir 2011, 27 (15), 9413–9417. 10.1021/la201535x. 21682338

[ref31] Wang H.; Liu Y.; Li J.; Luo J. Investigation of Superlubricity Achieved by Polyalkylene Glycol Aqueous Solutions. Adv. Mater. Interfaces 2016, 3 (19), 1600531 10.1002/admi.201600531.

[ref32] Li J.; Zhang C.; Luo J. Superlubricity Achieved with Mixtures of Polyhydroxy Alcohols and Acids. Langmuir 2013, 29 (17), 5239–5245. 10.1021/la400810c. 23597021

[ref33] Matta C.; Joly-Pottuz L.; De Barros Bouchet M. I.; Martin J. M.; Kano M.; Zhang Q.; Goddard W. A. Superlubricity and Tribochemistry of Polyhydric Alcohols. Phys. Rev. B Condens Matter Mater. Phys. 2008, 78 (8), 2–9. 10.1103/PhysRevB.78.085436.

[ref34] Ge X.; Li J.; Zhang C.; Liu Y.; Luo J. Superlubricity and Antiwear Properties of in Situ-Formed Ionic Liquids at Ceramic Interfaces Induced by Tribochemical Reactions. ACS Appl. Mater. Interfaces 2019, 11 (6), 6568–6574. 10.1021/acsami.8b21059. 30657308

[ref35] Ge X.; Li J.; Zhang C.; Wang Z.; Luo J. Superlubricity of 1-Ethyl-3-Methylimidazolium Trifluoromethanesulfonate Ionic Liquid Induced by Tribochemical Reactions. Langmuir 2018, 34 (18), 5245–5252. 10.1021/acs.langmuir.8b00867. 29672065

[ref36] Vlǎdescu S. C.; Tadokoro C.; Miyazaki M.; Reddyhoff T.; Nagamine T.; Nakano K.; Sasaki S.; Tsujii Y. Exploiting the Synergy between Concentrated Polymer Brushes and Laser Surface Texturing to Achieve Durable Superlubricity. ACS Appl. Mater. Interfaces 2022, 14 (13), 15818–15829. 10.1021/acsami.2c00725. 35333041 PMC9007417

[ref37] Reddyhoff T.; Ewen J. P.; Deshpande P.; Frogley M. D.; Welch M. D.; Montgomery W. Macroscale Superlubricity and Polymorphism of Long-Chain n-Alcohols. ACS Appl. Mater. Interfaces 2021, 13, 9239 10.1021/acsami.0c21918. 33565870

[ref38] Gohar R. Elastohydrodynamics; World Scientific: London, 2001.

[ref39] Spikes H.; Jie Z. History, Origins and Prediction of Elastohydrodynamic Friction. Tribol. Lett. 2014, 56 (1), 1–25. 10.1007/s11249-014-0396-y.

[ref40] Hamrock B. J.; Dowson D. Elastohydrodynamic Lubrication of Elliptical Contacts for Materials of Low Elastic Modulus. II - Starved Conjunction. *J. Lubrication Tech.*, 1979, 100( (1), ), 92–98 10.1115/1.3453284.

[ref41] Johnson K. L.; Tevaarwerk J. L. Shear Behaviour of Elastohydrodynamic Oil Films. Proc. R Soc. London Ser. A 1977, 356 (1685), 215–236. 10.1098/rspa.1977.0129.

[ref42] Eyring H. Viscosity, Plasticity, and Diffusion as Examples of Absolute Reaction Rates. J. Chem. Phys. 1936, 4 (4), 283–291. 10.1063/1.1749836.

[ref43] Zhang J.; Tan A.; Spikes H. Effect of Base Oil Structure on Elastohydrodynamic Friction. Tribol Lett. 2017, 65 (1), 1–24. 10.1007/s11249-016-0791-7.

[ref44] Yagi K. Influence of the Heat Transfer Eld on Anomalous Lubricant Lm Formation in Elastohydrodynamic Lubrication Conditions Tribol. Lett., 69 114 10.21203/rs.3.rs-500549/v1.

[ref45] Yagi K.; Vergne P. Abnormal Film Shapes in Sliding Elastohydrodynamic Contacts Lubricated by Fatty Alcohols. Proc. Inst. Mech. Eng., Part J 2007, 221 (3), 287–300. 10.1243/13506501JET253.

[ref46] Yagi K.; Vergne P. Film Thickness Changes in EHD Sliding Contacts Lubricated by a Fatty Alcohol 2006, 1, 5–8. 10.2474/trol.1.5.

[ref47] Yagi K.; Nishida K.; Sugimura J. Influence of the Heat Transfer Field on Anomalous Lubricant Film Formation in Elastohydrodynamic Lubrication Conditions. Tribol Lett. 2021, 69 (3), 114 10.1007/s11249-021-01492-0.

[ref48] Yagi K.; Nishida K.; Sugimura J. Relationship between the Molecular Structure of Lubricants and Appearance of Anomalous Film Shapes in Elastohydrodynamic Lubrication Conditions. Tribol Int. 2020, 152, 106574 10.1016/j.triboint.2020.106574.

[ref49] Yagi K.; Nishida K.; Sugimura J. Traction Behaviour of Elastohydrodynamic Lubrication Films with Anomalous Shapes. Proc. Inst. Mech. Eng., Part J 2021, 235 (11), 2247–2256. 10.1177/1350650121997240.

[ref50] Sirota E. B.; Wu X. Z. The Rotator Phases of Neat and Hydrated 1-Alcohols. J. Chem. Phys. 1996, 105 (17), 7763–7773. 10.1063/1.472559.

[ref51] Carreto L.; Almeida A. R.; Fernandws A. C.; Vaz W. L. C. Thermotropic Mesomorphism of a Model System for the Plant Epicuticular Wax Layer. Biophys. J. 2002, 82 (1), 530–540. 10.1016/S0006-3495(02)75418-0. 11751340 PMC1302493

[ref52] Zuo J.; Li W.; Weng L. Thermal Performance of Caprylic Acid/1-Dodecanol Eutectic Mixture as Phase Change Material (PCM). Energy Build 2011, 43 (1), 207–210. 10.1016/j.enbuild.2010.09.008.

[ref53] Wang P.; Reddyhoff T. Wall Slip in an EHL Contact Lubricated with 1-Dodecanol. Tribol Int. 2017, 113, 197–205. 10.1016/j.triboint.2016.10.034.

[ref54] Zhu H.; Dhinojwala A. Thermal Behavior of Long-Chain Alcohols on Sapphire Substrate. Langmuir 2015, 31 (23), 6306–6313. 10.1021/acs.langmuir.5b01330. 26010291

[ref55] Grunberg L.; Nissan A. H. Mixture Law for Viscosity. Nature 1949, 164, 799. 15395375 10.1038/164799b0

[ref56] Sathyanarayana B.; Ranjithkumar B.; Savitha Jyostna T.; Satyanarayana N. Densities and Viscosities of Binary Liquid Mixtures of N-Methylacetamide with Some Chloroethanes and Chloroethenes at T = 308.15 K. J. Chem. Thermodyn. 2007, 39 (1), 16–21. 10.1016/j.jct.2006.06.009.

[ref57] Woydt M.; Wäsche R. The History of the Stribeck Curve and Ball Bearing Steels: The Role of Adolf Martens. Wear 2010, 268 (11–12), 1542–1546. 10.1016/j.wear.2010.02.015.

[ref58] Guegan J.; Southby M.; Spikes H. Friction Modifier Additives, Synergies and Antagonisms. Tribol. Lett. 2019, 67 (3), 1–12. 10.1007/s11249-019-1198-z.

[ref59] Ishikawa M.; Yamamori K.; Hirano S.; Kowalski T.; Linden J. Introduction of Fuel Economy Engine Oil Performance Target with New SAE Viscosity Grade. SAE Int. J. Fuels Lubr. 2016, 9 (2), 374–382. 10.4271/2016-01-0896.

[ref60] Métivaud V.; Lefèvre A.; Ventolà L.; Négrier P.; Moreno E.; Calvet T.; Mondieig D.; Cuevas-Diarte M. A. Hexadecane (C16H34) + 1-Hexadecanol (C 16H33OH) Binary System: Crystal Structures of the Components and Experimental Phase Diagram. Application to Thermal Protection of Liquids. Chem. Mater. 2005, 17 (12), 3302–3310. 10.1021/cm050130c.

[ref61] Bair S. S.; Andersson O.; Qureshi F. S.; Schirru M. M. New EHL Modeling Data for the Reference Liquids Squalane and Squalane Plus Polyisoprene. Tribiol. Trans. 2018, 61 (2), 247–255. 10.1080/10402004.2017.1310339.

[ref62] Spikes H.; Jie Z. History, Origins and Prediction of Elastohydrodynamic Friction. Tribol. Lett. 2014, 1–25. 10.1007/s11249-014-0396-y.

[ref63] Pit R.; Hervet H.; Léger L. Direct Experimental Evidence of Slip in Hexadecane: Solid Interfaces. Phys. Rev. Lett. 2000, 85, 980 10.1103/PhysRevLett.85.980. 10991454

[ref64] Brmgman P.-W. Effects of High Shearing Stress Combined with High Hydrostatic Pressure. In Papers 94-121; Harvard University Press.

[ref65] Bair S. The Temperature Dependence of Divergence Pressure. Lubricants 2024, 12 (12), 434 10.3390/lubricants12120434.

[ref66] Choo J. H.; Forrest A. K.; Spikes Ha. Influence of Organic Friction Modifier on Liquid Slip: A New Mechanism of Organic Friction Modifier Action. Tribol Lett. 2007, 27 (2), 239–244. 10.1007/s11249-007-9231-z.

[ref67] Spikes H. Basics of EHL for Practical Application. Lubrication Sci. 2015, 27 (1), 45–67. 10.1002/ls.1271.

[ref68] Redlich O.; Kister A. T. Algebraic Representation of Thermodynamic Properties and the Classification of Solutions. Ind. Eng. Chem. 1948, 40 (2), 345–348. 10.1021/ie50458a036.

[ref69] McAllister R. A. The Viscosity of Liquid Mixtures. AIChE J. 1960, 6 (3), 427–431. 10.1002/aic.690060316.

[ref70] Cao W.; Knudsen K.; Fredenslund A.; Rasmussen P. Group-Contribution Viscosity Predictions of Liquid Mixtures Using UNIFAC-VLE Parameters. Ind. Eng. Chem. Res. 2088, 32, 2088–2092. 10.1021/ie00021a034.

[ref71] Montgomery W.; Zaug J. M.; Howard W. M.; Goncharov A. F.; Crowhurst J. C.; Jeanloz R. Melting Curve and High-Pressure Chemistry of Formic Acid to 8 GPa and 600 K. J. Phys. Chem. B 2005, 109 (41), 19443–19447. 10.1021/jp051967y. 16853512

[ref72] Vasileva A.; Golub P.; Doroshenko I.; Pogorelov V.; Sablinskas V.; Balevicius V.; Ceponkus J. FTIR Spectra of N-Octanol in Liquid and Solid States. Dataset Papers Sci. 2014, 2014, 921308 10.1155/2014/921308.

[ref73] Pocheć M.; Orzechowski K.; Rutkowski K. Indicators of Premelting in 1-Decanol and 1-Nonanol Studied by FTIR Spectroscopy. Surfaces Interfaces 2022, 28, 101676 10.1016/j.surfin.2021.101676.

[ref74] Zerbi G.; Conti G.; Minoni G.; Pisón S.; Bigotto A. Premelting Phenomena in Fatty Acids: An Infrared and Raman Study. J. Phys. Chem. A 1987, 2386–2393. 10.1021/j100293a038.

[ref75] Corsetti S.; Rabl T.; McGloin D.; Kiefer J. Intermediate Phases during Solid to Liquid Transitions in Long-Chain n-Alkanes. Phys. Chem. Chem. Phys. 2017, 19 (21), 13941–13950. 10.1039/C7CP01468F. 28513676 PMC6040280

[ref76] Socrates G. Infrared and Raman Characteristic Group Frequencies, 3rd ed.; John Wiley & Sons, 2001.

[ref77] Kamb W. B. Theory of Preferred Crystal Orientation Developed by Crystallization under Stress. J. Geol. 1959, 67 (2), 153–170. 10.1086/626571.

[ref78] Liu X.; Pulham C. R. Pressure-Induced Phase Separation of Miscible Liquids: 1:1: N-Pentane/Iso-Pentane. CrystEngComm 2020, 22 (47), 8251–8255. 10.1039/D0CE01335H.

[ref79] Boldyreva E. High-Pressure Polymorphs of Molecular Solids: When Are They Formed, and When Are They Not? Some Examples of the Role of Kinetic Control. Cryst. Growth Des. 2007, 7, 1662–1668. 10.1021/cg070098u.

[ref80] Dorighello Carareto N. D.; Costa M. C.; Meirelles A. J. A.; Pauly J. High Pressure Solid-Liquid Equilibrium of Fatty Alcohols Binary Systems from 1-Dodecanol, 1-Tetradecanol, 1-Hexadecanol, and 1-Octadecanol. J. Chem. Eng. Data 2015, 60 (10), 2966–2973. 10.1021/acs.jced.5b00330.

[ref81] Walton F.; Bolling J.; Farrell A.; MacEwen J.; Syme C. D.; Jiménez M. G.; Senn H. M.; Wilson C.; Cinque G.; Wynne K. Polyamorphism Mirrors Polymorphism in the Liquid-Liquid Transition of a Molecular Liquid. J. Am. Chem. Soc. 2020, 142 (16), 7591–7597. 10.1021/jacs.0c01712. 32249557 PMC7181258

[ref82] Keen D. A.; Goodwin A. L. The Crystallography of Correlated Disorder. Nature 2015, 303–309. 10.1038/nature14453. 25993960

[ref83] Reichenbach J.; Wynne K. Frustration vs Prenucleation: Understanding the Surprising Stability of Supersaturated Sodium Thiosulfate Solutions. J. Phys. Chem. B 2018, 122 (30), 7590–7596. 10.1021/acs.jpcb.8b04112. 29993246

[ref84] Jin B.; Zhang H.; Chen G.; Meng T.; Zhao J.; Zhang M.; Cao Y.; Fang D.; He Y.; Zhang C.; Yu X.; Zeng Q.; Luo J. Phase Transition Structural Superlubricity. Matter 2024, 7, 3107 10.1016/j.matt.2024.04.044.

[ref85] Mahajan A. R.; Mirgane S. R. Excess Molar Volumes and Viscosities for the Binary Mixtures of N-Octane, n-Decane, n-Dodecane, and n-Tetradecane with Octan-2-Ol at 298.15 K. Journal of Thermodynamics 2013, 2013 (1), 571918 10.1155/2013/571918.

[ref86] Arce A.; Rodil E.; Soto A. Volumetric and Viscosity Study for the Mixtures of 2-Ethoxy-2- Methylpropane, Ethanol, and 1-Ethyl-3-Methylimidazolium Ethyl Sulfate Ionic Liquid. J. Chem. Eng. Data 2006, 51 (4), 1453–1457. 10.1021/je060126x.

[ref87] Mahajan A. R.; Mirgane S. R. Excess Molar Volumes and Viscosities for the Binary Mixtures of N-Octane, n-Decane, n-Dodecane, and n-Tetradecane with Octan-2-Ol at 298.15 K. J. Thermodynamics 2013, 2013 (1), 571918 10.1155/2013/571918.

[ref88] Papaioannou D.; Bridakis M.; Panayiotou C. G. Excess Dynamic Viscosity and Excess Volume of JV-Butylamine + 1-Alkanol Mixtures at Moderately High Pressures. J. Chem. Eng. Data 1993, 38, 370–378. 10.1021/je00011a010.

[ref89] Yamaguchi T. Viscoelastic Relaxations of High Alcohols and Alkanes: Effects of Heterogeneous Structure and Translation-Orientation Coupling. J. Chem. Phys. 2017, 146 (9), 094511 10.1063/1.4977705.

[ref90] Bair S.; Habchi W. Quantitative Elastohydrodynamic Lubrication—Seventeen Years. J. Tribol 2024, 146 (8), 080801 10.1115/1.4065299.

[ref91] Mudge S. M. Fatty Alcohols-a Review of Their Natural Synthesis and Environmental Distribution. Soap Detergent Association 2005, 132, 1–141.

[ref92] www.futuremarketinsights.com/reports/friction-modifier-additives-market, 2023.

[ref93] Thakur D. S.; Kundu A. Catalysts for Fatty Alcohol Production from Renewable Resources. J. Am. Oil Chem.’ Soc. 2016, 1575–1593. 10.1007/s11746-016-2902-x.

[ref94] LaFountain A. R.; Johnston G. J.; Spikes H. A. The Elastohydrodynamic Traction of Synthetic Base Oil Blends. Tribiol. Trans. 2001, 44, 648–656. 10.1080/10402000108982506.

[ref95] Guo Y. B.; Liu C. R. Mechanical Properties of Hardened AISI 52100 Steel in Hard Machining Processes. J. Manuf. Sci. Eng. 2002, 124 (1), 1 10.1115/1.1413775.

[ref96] Ehret P.; Dowson D.; Taylor C. M. On Lubricant Transport Conditions in Elastohydrodynamic Conjuctions. Proc. R. Soc. A 1998, 454 (1971), 763–787. 10.1098/rspa.1998.0185.

[ref97] Johnston G. J.; Wayte R.; Spikes H. A. The Measurement and Study of Very Thin Lubricant Films in Concentrated Contacts. Tribiol. Trans. 1991, 34 (2), 187–194. 10.1080/10402009108982026.

[ref98] Sawada T.; Takemura K.; Kitamura K.; Kimura S. Crystal growth by pressure control using a diamond anvil cell. J. Cryst. Growth 1988, 88 (4), 535–536. 10.1016/0022-0248(88)90152-2.

