# Supplementary material for: Breaking the Tradeoff between Oil Film Thickness and Viscous Friction: n-Alcohol-Containing Lubricants in High-Pressure Contacts
Source: ACS Appl Mater Interfaces. 2025 Mar 17;17(12):19143–55. doi: 10.1021/acsami.4c22374 (PMC11955952; doi:10.1021/acsami.4c22374)
Supplement: Supplementary file 1 — am4c22374_si_001.pdf [file am4c22374_si_001.pdf]

Supporting Information for

Breaking the trade-off between oil film thickness and viscous friction – n-alcohol-containing lubricants in high-pressure contacts

Tom Reddyhoff,[1]*, Wren Montgomery,[2] Muhammad Aqif Suhaimee,[1]† Pushkar Deshpande,[1]† Yunhao Xia,[1]† Peng Wang,[1]† James Ewen[1]

[1]Department of Mechanical Engineering, Imperial College London, Exhibition Road, South Kensington, London, SW7 2AZ, United Kingdom.

[2]Science Innovation Platform, The Natural History Museum, Cromwell Road, South Kensington, London, SW7 5BD, United Kingdom.

*****Corresponding author. Email: t.reddyhoff@imperial.ac.uk

This document contains additional supporting data. Fig. S1 shows the elastohydrodynamic friction coefficient vs. maximum Hertz contact pressure for Squalane, dodecanol, and a 50:50 blend of the two. The graphs obtained using different specimen material combinations all coincide. The mixture graph has the same shape as the friction vs. pressure graph obtained for the mixture with the straight changed hexadecane in Fig 3. This suggests the dodecanol phase transitions from hexagonal to orthorhombic occur irrespective of the branching of the hydrocarbon molecule. Fig. S2 extends this by showing that similar phase-change behavior is observed when a commercial synthetic, polyalphaolefin, hydrocarbon base oil is used. Finally, Fig. S3 shows that alkanol-induced friction reduction can occur in a fully formulated oil, in this case when blended with the tetradecanol.

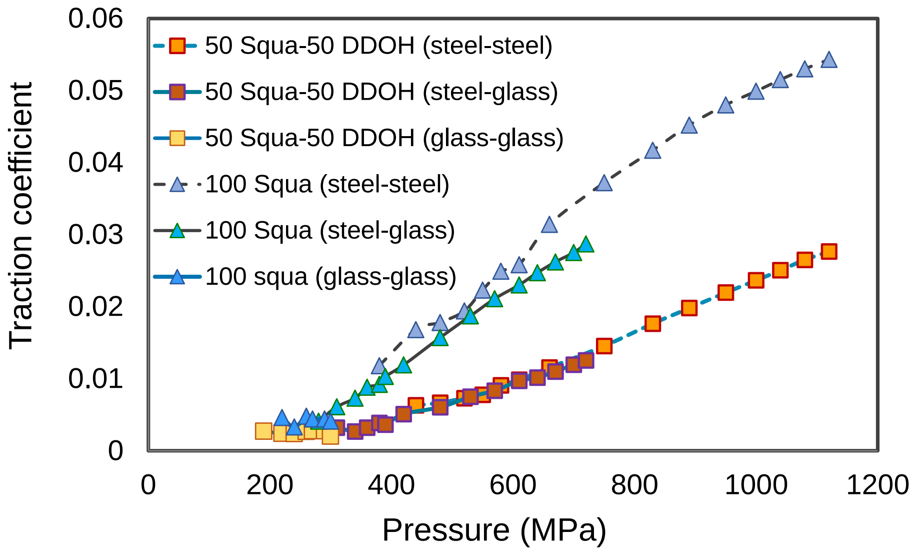

Fig. S1. EHL friction coefficient vs. maximum Hertz contact pressure for neat squalane and 50:50 blend of the squalane and dodecanol. The lubricant temperature is 40 °C, the slide-roll ratio is 30%, and the entrainment speed is 2 ms^{-1}.

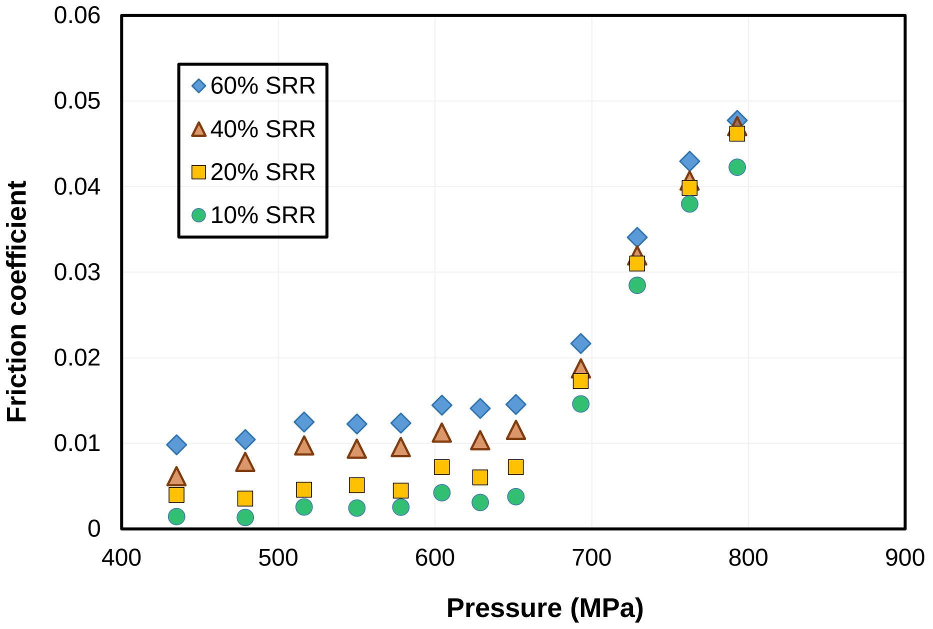

Fig. S2. EHL friction coefficient vs. maximum Hertz contact pressure for a 50:50 blend of the polyalphaolefin and dodecanol. The lubricant temperature is 50 °C, and the entrainment speed is 2 ms^{-1}.

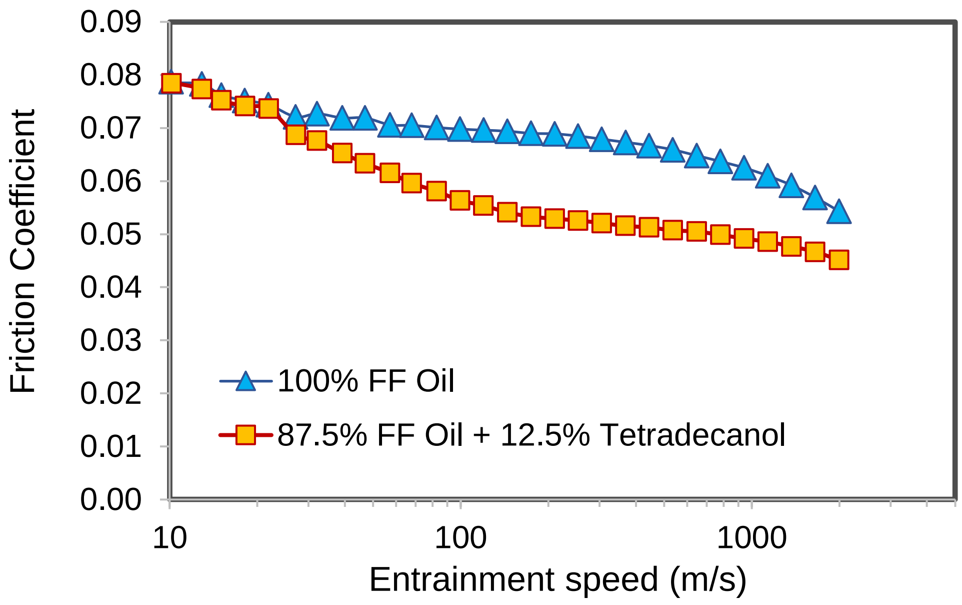

Fig. S3. EHL friction coefficient vs. entrainment speed for a fully formulated lubricant containing a commercial additive package, neat dodecanol, and a 50:50 blend of the two. The

applied load is 20 N, and the lubricant temperature is 40 °C. Here, the base oil components in the blend were adjusted so its viscosity equaled that of the unblended oil.
